# Full-length transcriptome sequencing reveals the low-temperature-tolerance mechanism of *Medicago falcata* roots

**DOI:** 10.1186/s12870-019-2192-1

**Published:** 2019-12-21

**Authors:** Guowen Cui, Hua Chai, Hang Yin, Mei Yang, Guofu Hu, Mingying Guo, Rugeletu Yi, Pan Zhang

**Affiliations:** 1 Department of Grassland Science, College of Animal Science and Technology, Northeast Agricultural University, Harbin, 150030 China; 2 Branch of Animal Husbandry and Veterinary of Heilongjiang Academy of Agricultural Sciences, Qiqihar, 161005 China; 3 Hulunbuir Grassland Station, Hulunbuir, 021008 China

**Keywords:** *Medicago falcata*, Low-temperature stress, SMRT, RNA-seq, WGCNA

## Abstract

**Background:**

Low temperature is one of the main environmental factors that limits crop growth, development, and production. *Medicago falcata* is an important leguminous herb that is widely distributed worldwide. *M. falcata* is related to alfalfa but is more tolerant to low temperature than alfalfa. Understanding the low temperature tolerance mechanism of *M. falcata* is important for the genetic improvement of alfalfa.

**Results:**

In this study, we explored the transcriptomic changes in the roots of low-temperature-treated *M. falcata* plants by combining SMRT sequencing and NGS technologies. A total of 115,153 nonredundant sequences were obtained, and 8849 AS events, 73,149 SSRs, and 4189 lncRNAs were predicted. A total of 111,587 genes from SMRT sequencing were annotated, and 11,369 DEGs involved in plant hormone signal transduction, protein processing in endoplasmic reticulum, carbon metabolism, glycolysis/gluconeogenesis, starch and sucrose metabolism, and endocytosis pathways were identified. We characterized 1538 TF genes into 45 TF gene families, and the most abundant TF family was the WRKY family, followed by the ERF, MYB, bHLH and NAC families. A total of 134 genes, including 101 whose expression was upregulated and 33 whose expression was downregulated, were differentially coexpressed at all five temperature points. PB40804, PB75011, PB110405 and PB108808 were found to play crucial roles in the tolerance of *M. falcata* to low temperature. WGCNA revealed that the MEbrown module was significantly correlated with low-temperature stress in *M. falcata*. Electrolyte leakage was correlated with most genetic modules and verified that electrolyte leakage can be used as a direct stress marker in physiological assays to indicate cell membrane damage from low-temperature stress. The consistency between the qRT-PCR results and RNA-seq analyses confirmed the validity of the RNA-seq data and the analysis of the regulatory mechanism of low-temperature stress on the basis of the transcriptome.

**Conclusions:**

The full-length transcripts generated in this study provide a full characterization of the transcriptome of *M. falcata* and may be useful for mining new low-temperature stress-related genes specific to *M. falcata*. These new findings could facilitate the understanding of the low-temperature-tolerance mechanism of *M. falcata.*

## Background

Low temperature is one of the main environmental factors that limit plant growth, development and geographical distribution [1]. Low-temperature stress, which consists of chilling stress (< 10 °C) and freezing stress (< 0 °C), can reduce crop productivity to some extent [2]. The effects of low temperature on plants depend on developmental stage and exposure time. Under low-temperature stress, young tissues and organs are more severely damaged than old tissues and organs, and the reproductive stage is more sensitive to low temperature than the vegetative stage is [3]. Exposure to low temperature causes physiological and molecular changes in plants that lead to metabolic disorders: these changes include an inhibition of photosynthetic activity; a reduction in water uptake; an increase in oxidative stress via increased accumulation of reactive oxygen species (ROS); an increase in intracellular pH and osmotic pressure; and functional abnormalities in chloroplasts, mitochondria and other organelles [4–6]. Furthermore, low temperature temporarily inhibits sucrose synthesis, and the rearrangement of membranes changes the stability and mobility of proteins and a shift in redox homeostasis, which decrease enzyme activities and alters both metabolic homeostasis and gene transcription [7, 8].

Plants have developed many mechanisms and pathways that enable them to minimize the negative effects of low temperature and to successfully grow and reproduce [9]. Osmolytes induced by low-temperature stress, including proline, soluble sugars, and cold-induced stress proteins (dehydrins and LEA proteins), can improve the osmotic potential of cells, protect the stability of biological membranes, alleviate oxidative damage limitations, and even act as signals to regulate the expression of stress-related genes [10, 11]. The overexpression of *SlNAM1*, which encodes a typical NAC, improves low-temperature tolerance in transgenic tobacco by improving osmolytes and reducing the H_2_O_2_ and superoxide anion radical (O_2_^.-^) contents under low temperature, which contribute to alleviating the oxidative damage of the cell membrane after low-temperature stress [12]. Low temperature induces the production of Ca^2+^, which can be sensed by corresponding receptors, among which lipid Ca^2+^ channels may be the primary cryogenic signal receptors, and can then activate calcium response protein kinase (CPKs, CIPKs, and CRLK1) and MAPK cascade responses, which in turn regulate the expression of cold-responsive (COR) genes [13, 14]. The overexpression of *COLD1* (jap) significantly increases chilling tolerance; *COLD1* interacts with the G-protein alpha subunit to activate Ca^2+^ channels to sense low temperature and to accelerate G-protein GTPase activity [15]. Low-temperature stress rapidly induces the expression of many transcription factors (TFs), including CBF AP2-domain proteins, which then activate the expression of numerous downstream *COR* genes [16–18]. The expression of the *CBF* gene is controlled by upstream TFs, such as the bHLH TF *ICE1*. *ICE1* is subjected to sumoylation and polyubiquitylation and subsequent proteasomal degradation mediated by the SUMO E3 ligase SIZ1 and the ubiquitin E3 ligase HOS1, respectively [13, 16].

Single-molecule long-read (SMRT) sequencing, which was developed by PacBio Biosciences RSII, represents a third-generation sequencing platform. Owing to its long reads, this platform is widely used in genome sequencing, and by generating full-length or long sequences, it has eliminated many restrictions associated with sequencing [19–22]. Next-generation sequencing (NGS) technology (RNA sequencing [RNA-seq]) can provide expression profiles of both coding or noncoding RNAs and can generate digital data of gene expression, enabling rapid and cost-effective genomic and transcriptomic studies for most major crop species, including rice [23], wheat [24], and grape [25]. The approach by which NGS and SMRT sequencing are combined has been applied frequently to generate comprehensive information at the transcriptional level, allowing the identification of key functional and regulatory genes involved in abiotic stress resistance [26–28]. The changes in gene expression in response to low temperature stress revealed by transcriptome analyses including TFs, protein kinases, small non-coding RNAs and enzymes in metabolic pathways, which regulated the expression of downstream genes, target genes, and protected cells from low-temperature damage [29, 30].

*Medicago falcata* L., an economically and ecologically important legume herb with an expanse from northern Mediterranean regions to northern Russia, is closely related to alfalfa but exhibits better tolerance to low temperature than alfalfa [31–33]. Understanding the mechanism of low-temperature tolerance in *M. falcata* is important for the genetic improvement of alfalfa, which is the most important forage leguminous species because of its high biomass productivity, optimal nutritive profile and adequate persistence [34]. Although the low-temperature tolerance of *M. falcata* is a popular research topic at the morphological level to the physiological, biochemical and molecular biology levels [31, 35–37], few studies have investigated the low-temperature tolerance of *M. falcata* at the transcriptome level. In this paper, we combined SMRT and NGS to generate expression profiles of *M. falcata* roots under low-temperature stress. In total, 115,153 nonredundant sequences were obtained from *M. falcata* roots, and 11,369 differentially expressed genes (DEGs) were identified, including 134 genes that were differentially coexpressed at all five temperature points. These findings provide a global characterization of gene transcription and facilitate the understanding of the low-temperature-tolerance mechanisms of *M. falcata.*

## Results

### Physiological responses of *M. falcata* under low-temperature stress

**Fig. 1:**
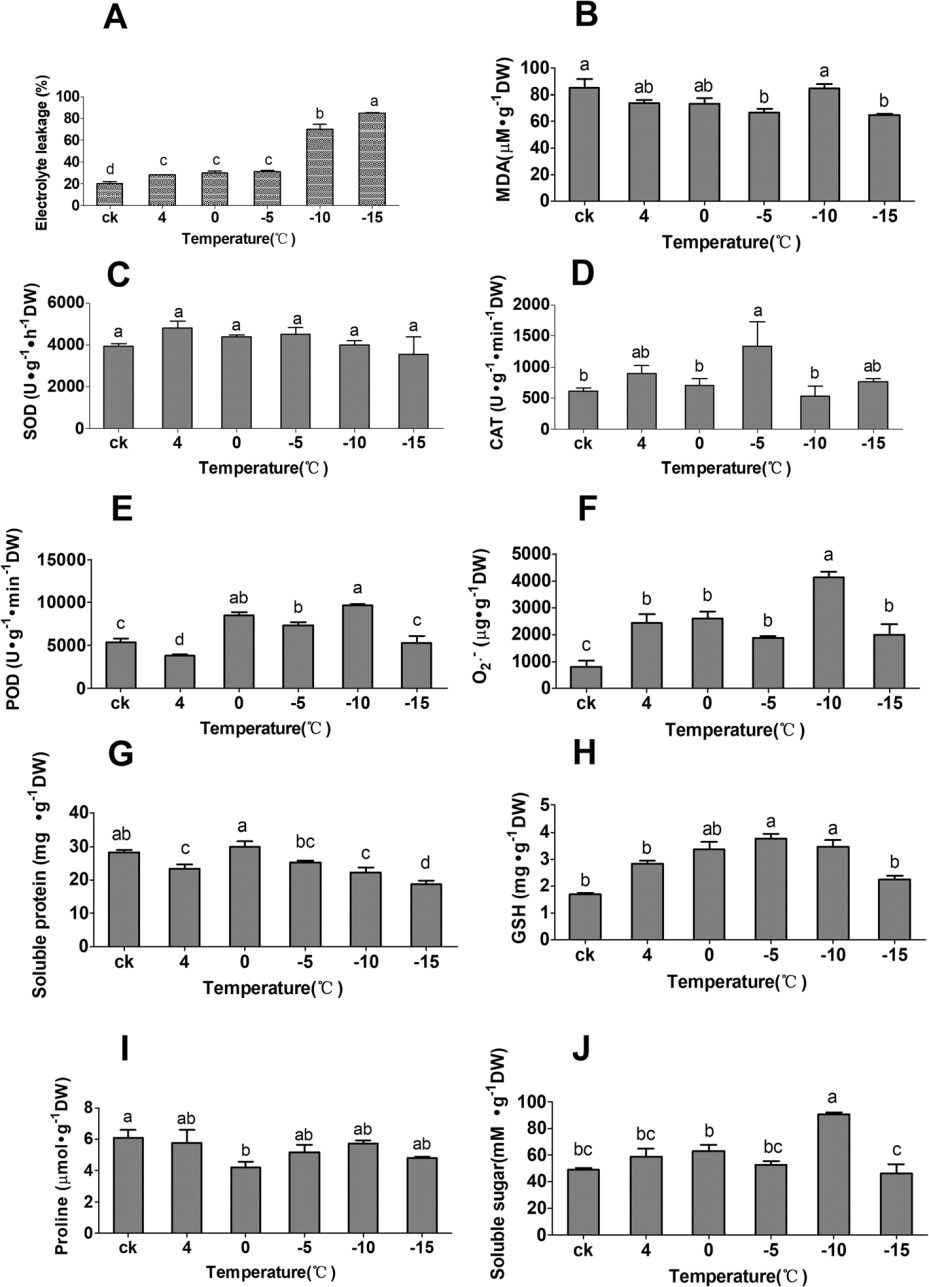
Determination of physiological indices of the roots of *M. falcata* plants under low-temperature stress. **a**, Electrolyte leakage. **b**, MDA content. **c**, SOD activity. **d**, CAT activity. **e**, POD activity. **f**, O_2_^.-^ content. **g**, Soluble protein content. **h**, GSH content. **i**, Proline content. **j**, Soluble sugar content. The data are shown as the means ± SDs of four independent experiments. The different letters represent statistically significant differences as determined by one-way ANOVA (*p* < 0.05, Duncan’s multiple range test)

We measured the electrolyte leakage, malondialdehyde (MDA) content, superoxide dismutase (SOD), catalase (CAT) and peroxidase (POD) activities as well as the superoxide anion radical (O_2_^.-^), soluble protein, reduced glutathione (GSH), proline and soluble sugar contents to investigate the physiological changes in *M. falcata* roots exposed to low-temperature stress for 2 h (Fig. 1). Under low-temperature stress, the electrolyte leakage increased gradually with decreasing temperature (Fig. 1a). The greatest electrolyte leakage was observed at − 15 °C, which was 4.18 times greater than that under the control conditions. The MDA content decreased gradually, peaked at − 10 °C (relative to that of the control) and slightly decreased at − 15 °C (Fig. 1b). No obvious difference in SOD activity was observed, and the activity increased after low-temperature treatment and decreased from 4 °C to − 15 °C (Fig. 1c). The changes in CAT and POD activities were completely different under different temperatures. CAT activity first increased, whereas the POD activity first decreased (Fig. 1d-e). The content of O_2_^.-^ increased significantly after low-temperature treatment and peaked at − 10 °C (Fig. 1f). The content of soluble protein decreased first after low-temperature treatment but then increased after 4 °C, after which it decreased significantly after 0 °C (Fig. 1g). The GSH content increased significantly after low-temperature treatment and peaked at − 5 °C (Fig. 1h). The proline content decreased slightly in the low-temperature environment (Fig. 1i), and the greatest soluble sugar content was measured at − 10 °C (Fig. 1j).

### *M. falcata* transcriptome sequencing

To identify and characterize the transcriptome of *M. falcata* roots under low-temperature stress, we measured the roots under room temperature (CK), 4 °C, 0 °C, − 5 °C, − 10 °C and − 15 °C for 2 h by combining the PacBio SMRT and NGS technologies for whole-transcriptome profiling. In total, 125.48 Gb of clean data were obtained by RNA-seq, yielding 418,495,643 reads (with a GC content of 42.61% and a QC 30 of 87.55%) (Additional file 1: Table S1). A total of eight SMRT cells were used for the three libraries at three size ranges, 1–2 kb, 2–3 kb, and 3–6 kb, yielding 19.27 Gb of clean data. A total of 1,202,336 polymerase reads were obtained, and the polymerase reads containing fragments that were less than 50 bp in length and with a sequence accuracy lower than 0.75 were subsequently filtered and removed. A total of 8,428,385 subreads were then obtained by filtering the remaining sequences from the linkers, the linker sequences and the subreads with fragments less than 50 bp in length (Table 1). A total of 552,818 reads of inserts (ROIs, of which 270,750 were full-length nonchimeric reads (FLNCs), and 223,319 were non-full-length reads, were extracted from the original sequence (Table 1). The full-length sequences were clustered via the RS_IsoSeq module of SMRT Analysis software. A total of 131,118 consensus isoforms were obtained; 99,490 high-quality isoforms were obtained via non-full-length sequence alignment, and 31,628 low-quality isoforms were obtained and corrected via RNA-seq data. Any redundancy within the high-quality and corrected low-quality transcript sequences of each sample was eliminated by CD-HIT software, and 115,153 nonredundant sequences were obtained. On the basis of the nonredundant sequences of each sample, we predicted a total of 8849 alternative splicing (AS) events via the IsoSeq_AS_de_novo script (Additional file 2: Table S2); 73,149 simple sequence repeats (SSRs) via the MIcroSAtellite identification tool (Additional file 3: Table S3); and 4189 long noncoding RNAs (lncRNAs) with the coding potential calculator (CPC), coding–non-coding index (CNCI), coding potential assessment tool (CPAT), and Protein family (Pfam) database information (Additional file 4: Figure S1).

**Table 1:** Statistical results of the SMRT sequencing data

cDNA size	1–2 kb	2–3 kb	> 3 kb	All
SMRT cells	3	3	2	8
Polymerase reads	450,876	450,876	300,584	1,202,336
Postfilter number of subreads	4,761,465	2,481,575	1,185,345	8,428,385
Reads of insert	240,441	189,779	122,598	552,818
Number of 5′ reads	122,516	120,763	84,001	327,280
Number of 3′ reads	139,768	126,512	87,086	353,366
Number of poly-A reads	133,709	124,866	86,112	344,687
Number of filtered short reads	42,363	11,932	2853	57,148
Number of non-full-length reads	100,142	75,634	47,543	223,319
Number of full-length reads	97,936	102,213	72,202	272,351
Number of full-length nonchimeric reads	97,156	101,959	71,635	270,750
Average full-length nonchimeric read length	1309	2282	3536	2264
Full-length percentage (FL%)	40.73%	53.86%	58.89%	49.27%
Artificial concatemers (%)	0.80%	0.25%	0.79%	0.59%

### Annotation and expression of transcripts under low-temperature stress

To acquire the most comprehensive annotation information, all full-length transcripts from SMRT were aligned with NCBI nonredundant protein (Nr), SwissProt, Gene Ontology (GO), Clusters of Orthologous Groups (COG), Eukaryotic Ortholog Groups (KOG), Pfam, and Kyoto Encyclopedia of Genes and Genomes (KEGG) database information via BLAST software (version 2.2.26), and a total of 111,587 genes from SMRT were annotated, of which the length of 7155 genes was > = 300 bp and < 1000 bp and of which the length of 104,432 genes was > = 1000 bp (Additional file 5: Table S4). Among these annotated sequences, 111,384 sequences had significant matches in the Nr database, 89,117 sequences had significant matches in the Pfam database, 82,433 sequences had matches in the SwissProt database, and 3385 had effective matches in the GO database. On the basis of the homology among sequences of different species, 97,831 (87.87%) sequences were found against *M. truncatula*, and 3463 (3.11%) sequences had clandestine hits with *Cicer arietinum*, followed by *M. sativa* (1662, 1.49%), *Glycine max* (1011, 0.91%), *Rhizoctonia solani* (528, 0.47%), *Fusarium oxysporum* (388, 0.35%), *Glycine soja* (369, 0.33%), *Phaseolus vulgaris* (349, 0.31%), *Vitis vinifera* (266,0.24%), and *M. falcata* (181, 0.16%). Only 5283 (4.75%) annotated sequences were similar to those of other plant species (Additional file 6: Figure S2).

**Fig. 2:**
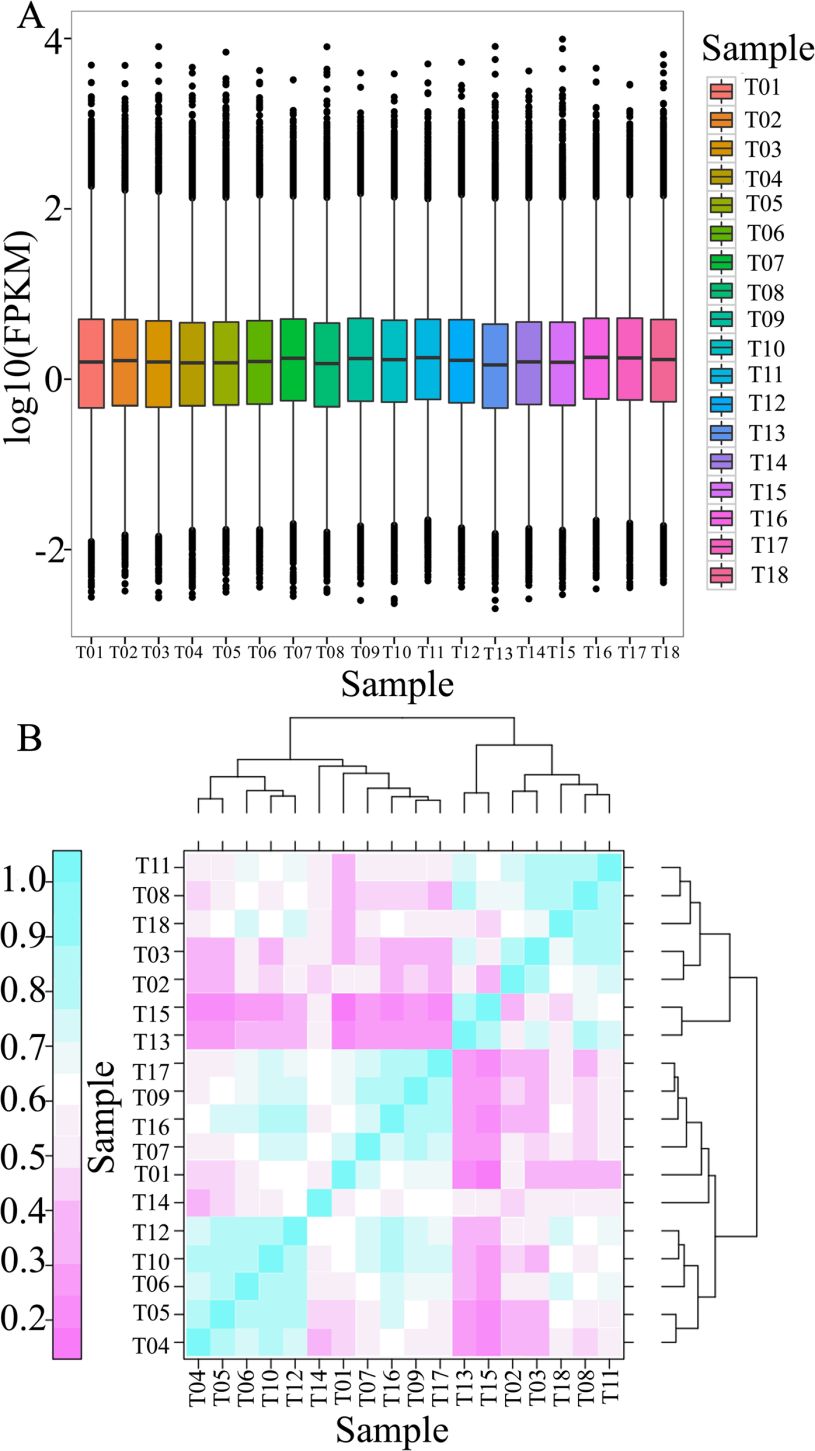
Comparison of gene expression levels under low-temperature stress. **a**, Boxplot showing the distribution of the FPKM values of each sample under low-temperature stress. The X-axis in the boxplot shows the ID of each sample. The Y-axis represents the log_10_(FPKM). **b**, Heat map of the Pearson correlation coefficient of each sample under low-temperature stress

To evaluate gene expression levels in response to low-temperature stress, we mapped all the clean data back to the assembled transcriptome, and the read count for each gene was obtained from the mapping results via RNA-seq by expectation maximization (RSEM) software (Additional file 7: Table S5). The mapped read count for each gene was then converted to the expected number of fragments per kilobase of transcript per million mapped reads (FPKM) (Additional file 8: Table S6). FPKM values can eliminate the effects of transcript length and sequencing differences on computational expression. A boxplot diagram of the FPKM values indicated that gene expression levels were not evenly distributed in the different experimental environments (Fig. 2a). Pearson correlation coefficients were subsequently used to evaluate the correlations of each biological sample, with r^2^ values close to 1 indicating a strong correlation between two replicate samples (Fig. 2b). Afterward, all the sequences were then used for further DEG analysis after abnormal samples were excluded.

### Analysis of DEGs in response to low-temperature stress

**Fig. 3:**
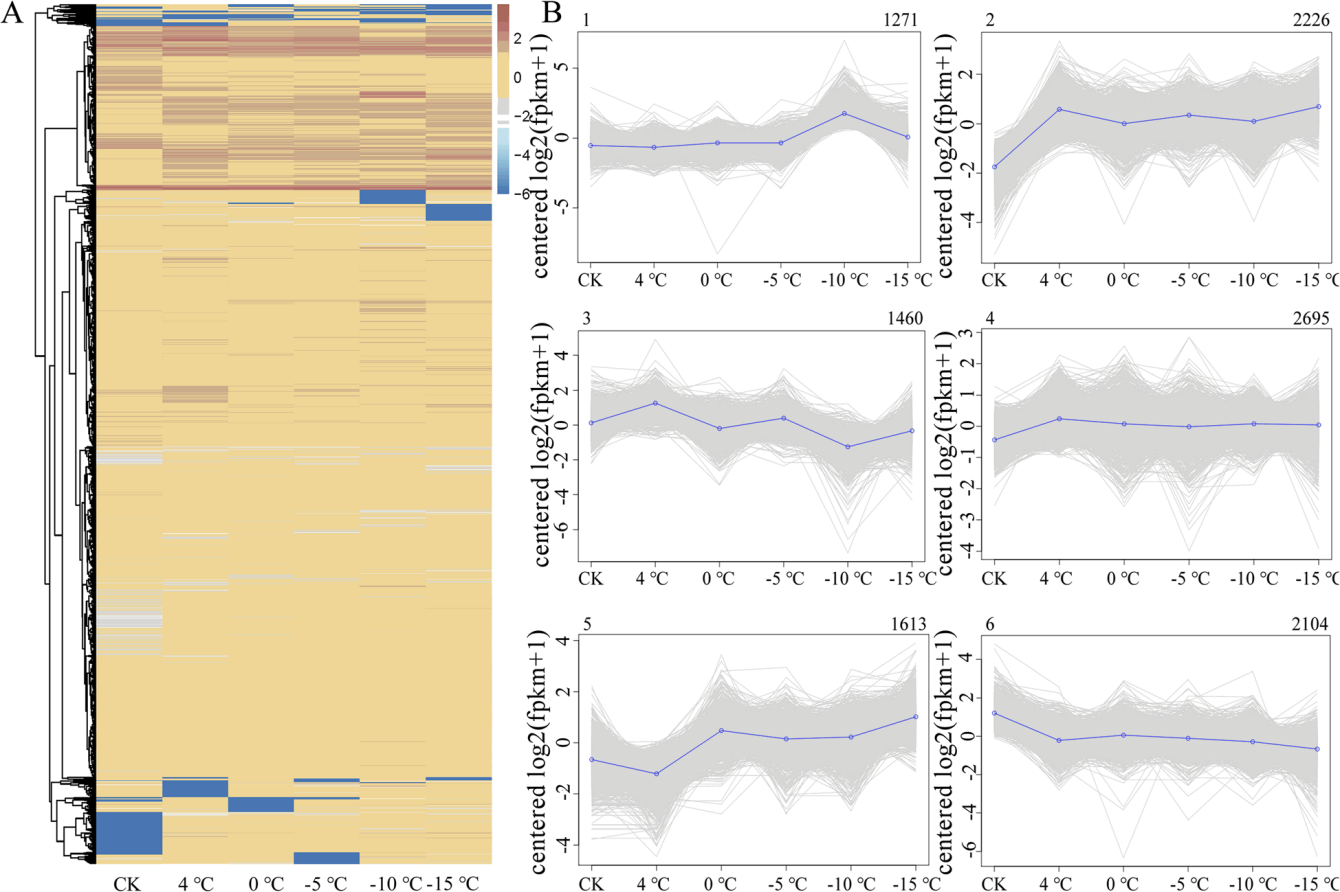
Clustering analysis of the DEGs. **a**, Hierarchical clustering of the 11,369 DEGs on the basis of the averaged log_2_(FPKM+ 1) values of all genes in each cluster. **b**, The six subclusters of the 11,369 DEGs were clustered. The number of genes in each subcluster is shown at the top of the subcluster. The blue line shows the average values of the relative expression levels in each subcluster, and the gray lines represent the relative gene expression levels of each gene in each subcluster

In total, 11,369 DEGs whose expression was up- or downregulated between samples (fold change≥2 and false discovery rate (FDR) < 0.01) at any pair of temperature points were identified by comparing gene expression levels under low-temperature stress (Additional file 9: Table S7). Clustering patterns of the DEGs of plants under low-temperature stress were determined by hierarchical cluster analysis of all the DEGs (Fig. 3a). All DEGs exhibiting the same or similar expression levels were clustered, and it was determined that a set of genes was quickly expressed during the early stage of low-temperature stress (4 °C) and that other genes were expressed under freezing temperature (− 10 °C). The 11,369 DEGs identified were grouped into six subclusters by K-means coexpression cluster analysis (Fig. 3b). The expression level of genes in subcluster 1 (1271 genes) began to increase after the temperatures decreased to − 5 °C and reached a maximum at − 10 °C; the expression levels then decreased rapidly. KEGG analysis of the genes in subcluster 1 revealed that most were involved in starch and sucrose metabolism, plant-pathogen interactions, and galactose metabolism. The expression of genes in subcluster 2 (2226 genes) increased significantly after low-temperature treatment, after which the level first decreased slowly and then rapidly at each temperature point below 4 °C. Genes in this subcluster functioned mostly in the circadian rhythm, plant-pathogen interactions, and plant hormone signal transduction. The expression of the genes in both subcluster 3 (1460 genes) and subcluster 4 (2695 genes) was upregulated after low-temperature treatment and downregulated after 4 °C treatment. The difference was that the decrease in the expression of genes in subcluster 3 fluctuated, while that in subcluster 4 was continuous. Genes involved in starch and sucrose metabolism, protein processing in endoplasmic reticulum, fatty acid degradation, and tyrosine metabolism were enriched in subcluster 3, and genes in subcluster 4 were enriched in plant-pathogen interactions, starch and sucrose metabolism and plant hormone signal transduction. The expression of genes in subcluster 5 (1613 genes) was weakly downregulated after low-temperature treatment, strongly upregulated from 4 °C to 0 °C, and then upregulated again under − 15 °C treatment. The genes in this subcluster functioned mostly in plant-pathogen interactions, phenylalanine metabolism, and the plant hormone signal transduction pathway. The expression of genes in subcluster 6 (2104 genes) was downregulated at all times, and most of these genes functioned in phenylpropanoid biosynthesis, circadian rhythm, and ubiquitin-mediated proteolysis.

### Identification of putative TFs

TFs play an important role in cell function and development and directly regulate gene expression via interactions with themselves and other proteins to participate in plant stress regulation, including low-temperature-related processes. In this study, 1538 TF genes were differentially expressed between different temperature points and were classified into 45 TF gene families according to information in the PlantTFDB (http://planttfdb.cbi.pku.edu.cn/) (Additional file 10: Table S8). The most abundant TF family was the WRKY (186 genes) family, followed by the ERF (165 genes), MYB (143 genes), bHLH (131 genes) and NAC (111 genes) families. The cluster analysis of TF gene expression indicated that the expression of some of these TF genes was extensively upregulated in response to low temperature (Additional file 11: Figure S3), such as those encoding bZIP, AP2/ERF, MYB, C2H2 and WRKY and TFs.

### Comparison of coexpressed genes between low-temperature-treated samples and control samples

**Fig. 4:**
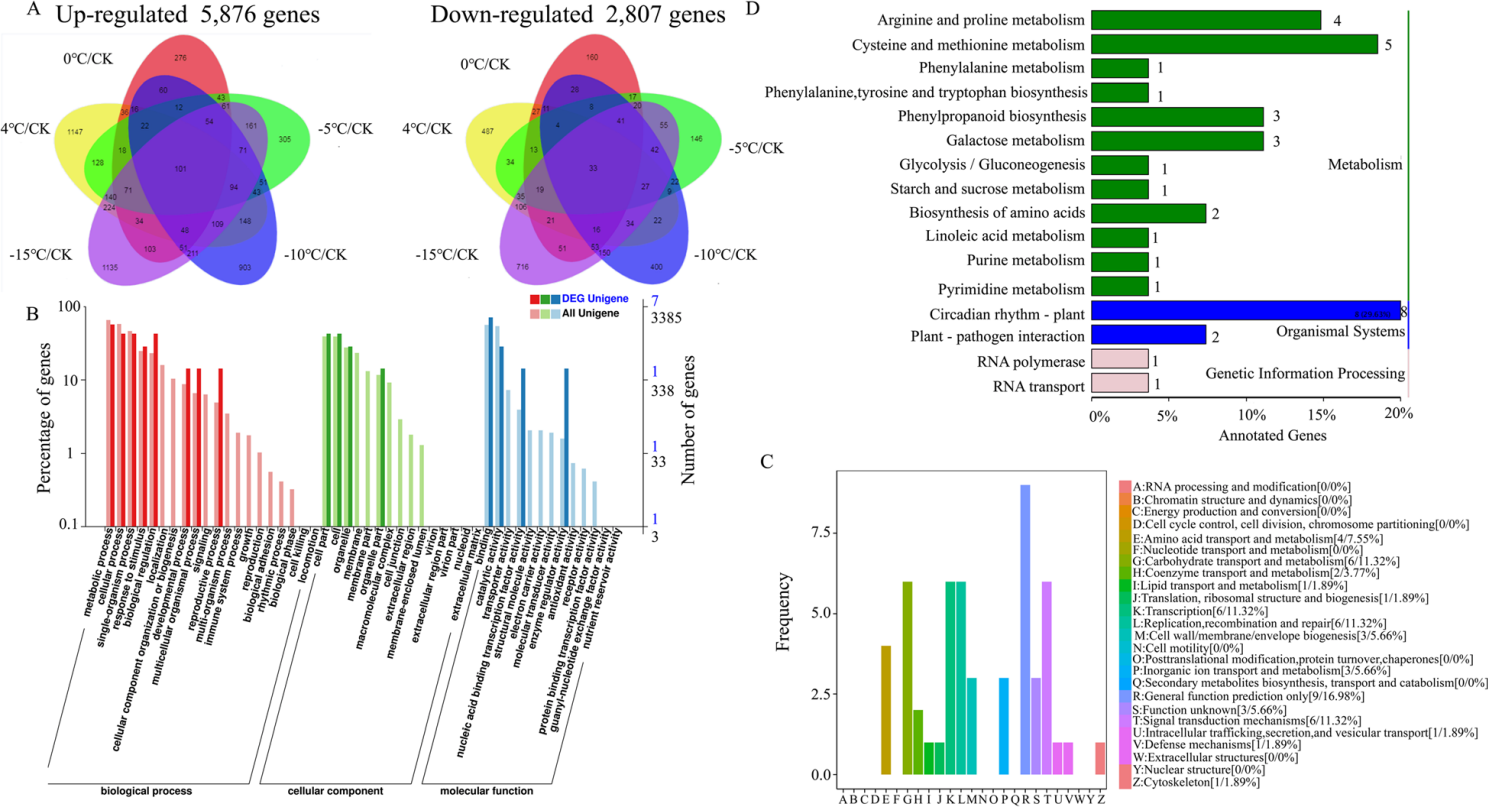
Summary of coexpressed genes between low-temperature-treated samples and control samples. **a**, Venn diagram of DEGs identified by a comparison of each temperature point to the control environment. **b**, GO classification of 134 coexpressed genes. **c**, COG functional classification of 134 coexpressed genes. **d**, KEGG enrichment analysis of 134 coexpressed genes

Under low-temperature stress, a total of 8683 DEGs were identified by a comparison of each temperature point to the control environment. Interestingly, there were many more upregulated DEGs (5876 genes) than downregulated DEGs (2807 genes), and 134 genes were differentially coexpressed at all five temperature points (Additional file 12: Table S9). As shown in Fig. 4a, there were 101 upregulated genes and 33 downregulated genes across all five comparisons. These 134 coexpressed genes were assigned to the biological process, cellular component and molecular function GO categories (Fig. 4b). In the biological process category, “metabolic process”, “cellular process”, “single-organism process” and “biological regulation” were the most enriched terms. In the cellular component category, “cell”, “cellular component” and “organelle” were the most enriched, and in the molecular function category, “binding” was the most enriched term, followed by “catalytic activity”. COG functional classification of the 134 coexpressed genes showed that most of the genes were enriched in “general function prediction only”, “transcription”, “replication, recombination and repair”, “signal transduction mechanisms” and “carbohydrate transport and metabolism” (Fig. 4c). KEGG enrichment revealed that most of the coexpressed genes were enriched in the “circadian rhythm - plant”, “cysteine and methionine metabolism”, “arginine and proline metabolism”, “phenylpropanoid biosynthesis”, “galactose metabolism”, “plant-pathogen interaction” and “biosynthesis of amino acids” pathways (Fig. 4d).

### Weighted gene coexpression network analysis (WGCNA) of DEGs in response to low-temperature stress

**Fig. 5:**
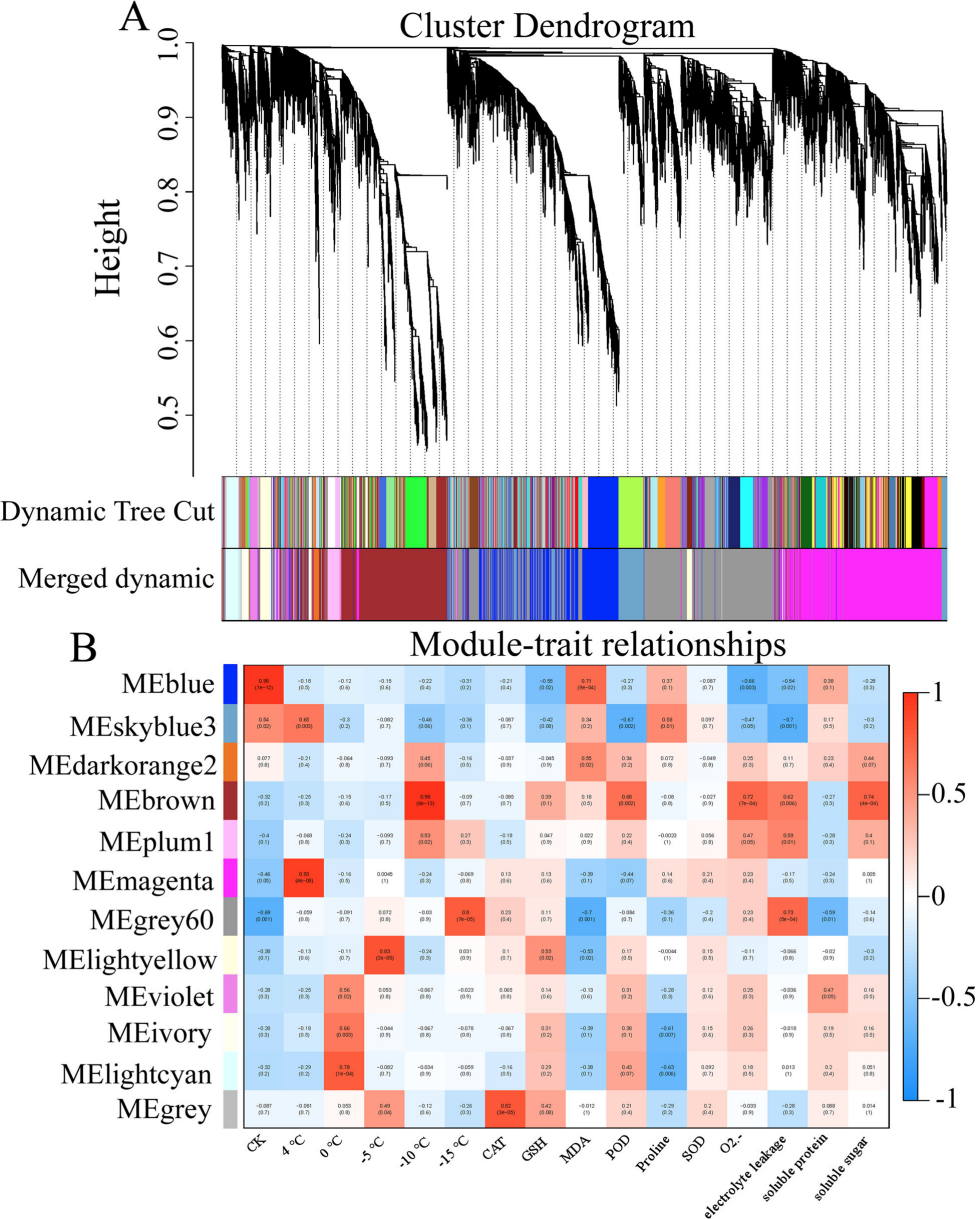
WGCNA of the genetic modules related to each sample and physiological indicators. **a**, Cluster dendrogram. **b**, Module–trait relationships

WGCNA was performed to better understand which genes within these complex signaling networks were the most connected hubs. The number of genes in the module were clustered according to their expression levels, and those genes with a high clustering degree were allocated to the same models. The 8683 DEGs identified by comparing low-temperature-treated samples and control samples were clustered on the basis of topological overlap, and then the gene modules were obtained from a dynamic tree cut. Finally, 12 gene modules were identified after merging modules whose expression patterns were similar (Fig. 5a). The magenta modules contained the most genes (1092), and the violet modules contained the fewest (70) (Additional file 13: Table S10). The gray module was not a true module but a place to categorize the remaining genes that were not well correlated with any one of the significant colored modules. The module eigengene-based connectivity (kME) value was calculated for each gene to every module, and 449 genes were found to act as a hub in more than one module.

All 12 genetic modules with a module characteristic value of *p* < 0.05 were used to identify the modules that were highly correlated with samples and physiological indicators (Fig. 5b). The control samples were highly correlated with the MEblue module. The samples under 4 °C treatment were highly correlated with the MEmagenta module, and the samples under 0 °C treatment were highly correlated with the MElightcyan module. The samples under − 5 °C treatment were highly correlated with the MElightyellow module, the samples under − 10 °C treatment were highly correlated with the MEbrown module, and the samples under − 15 °C treatment were highly correlated with the MEgray60 module. The MEbrown module was found to be significantly correlated with physiological indicators and may play a key role in the low-temperature tolerance of *M. falcata*. The COG classification results showed that the MEbrown module genes were involved in 23 major categories, including “general function prediction only”, “signal transduction mechanisms”, “transcription” and “replication, recombination and repair” (Additional file 14: Figure S4A). The GO analysis results showed that the MEbrown module genes were involved mainly in 13 biological processes, including “protein phosphorylation”, “regulation of transcription, DNA-templated”, and “oxidation-reduction process”. These genes were distributed to eight cellular component terms, including “integral component of membrane”, “plasmodesma”, “chloroplast stroma” and “nucleus”, and were associated with 14 molecular functions, which included ATP binding, protein serine/threonine kinase activity, and cation binding (Additional file 14: Figure S4B). KEGG enrichment analysis showed that most of the genes in the MEbrown module were enriched in “starch and sucrose metabolism”, “plant-pathogen interaction”, “circadian rhythm - plant”, “protein processing in endoplasmic reticulum”, “galactose metabolism” and “plant hormone signal transduction” (Additional file 14: Figure S4C).

### Confirmation of RNA-seq sequencing data by qRT-PCR analysis

The DEGs associated with low-temperature stress were selected for qRT-PCR assays to confirm the SMRT sequencing data. Twenty genes were selected randomly from 134 DEGs coexpressed at all five temperature points. We found that the fold-changes in expression calculated via the sequencing data did not exactly match the expression values detected by qRT-PCR analysis, but the expression profiles were essentially consistent for all 20 genes (Additional file 15: Figure S5). These analyses confirmed the reliability of the gene expression values generated from the SMRT sequencing data.

## Discussion

The approach of combining NGS and SMRT sequencing has become increasingly popular for studying plant responses to adverse environmental conditions to provide high-quality and increasingly complete assemblies at the transcriptome level [26–28]. SMRT sequencing can generate full-length or long sequences, and the high error rate can be overcome via short and high-accuracy NGS reads [19, 22]. In this study, we combined the SMRT and RNA-seq methods to analyze the transcriptome assembly of roots of *M. falcata* plants under low-temperature stress and to identify key functional and regulatory genes involved in low-temperature tolerance. We ultimately obtained 115,153 nonredundant sequences, and the average ROI was long enough to represent the full-length transcripts (Table 1). We also predicted a total of 8849 AS events, 73,149 SSRs, and 4189 lncRNAs.

### Changes in physiological indicators

Our results indicated the complexity of physiological changes within *M. falcata* plants in response to low-temperature stress. Under low-temperature stress, the roots displayed relatively more extensive changes in membranes, antioxidants and osmolytes. An increase in the contents of MDA, proline, and soluble sugars as well as electrolyte leakage has been demonstrated in cold-treated wheat and wild tomato [25, 38]. During low-temperature treatment, *M. falcata* accumulates relatively high amounts of sucrose and proline and exhibits high sucrose phosphate synthase (SPS) and sucrose synthase activity [39]. In this paper, an increase in MDA, proline and soluble sugar contents as well as electrolyte leakage was observed in the roots of low-temperature-treated *M. falcata* plants, suggesting that osmolytes might protect plant cell membranes, increase membrane stabilization, and balance osmotic pressure during low-temperature-induced dehydration of *M. falcata*; additionally, electrolyte leakage can be used as a direct stress marker to reflect cell membrane damage by low-temperature stress (Fig. 1). Low-temperature stress induces the activities of cell apoptosis factors, and it has been proven that injury to *M. falcata* under low temperature is related to proteins in cells [40]. The enzymatic antioxidant system is a protective mechanism used to eliminate or reduce ROS and increase a plant’s ability to tolerate low-temperature stress [41, 42]. Thus, the changes in CAT, POD, and GSH may play a key role in the detoxification of ROS induced by low temperature in *M. falcata* roots.

### Gene expression in the roots of *M. falcata* in response to low-temperature stress

In this study, a total of 11,369 DEGs were identified as responsive to low-temperature stress at all temperature points, of which the expression of 68.8% was induced and that of 31.2% was repressed under low-temperature stress. All DEGs were grouped into six subclusters (Fig. 3b), after which KEGG pathway enrichment analysis was conducted. In the organismal systems category, the most enriched pathway was “plant-pathogen interaction”, indicating a basic plant immunological response in the roots of *M. falcata* plants under low-temperature stress [43]. In the category of environmental information processing, most DEGs were involved in the plant hormone signal transduction pathway. Our results demonstrated the critical role of phytohormones in plants in response to external and internal cues to regulate growth and development [36]. In the genetic information processing category, the most enriched pathway was “protein processing in endoplasmic reticulum”. Under low-temperature stress, the membrane protein synthesis rate and membrane protein number increased in cold-adapted alfalfa [44]. Our data showed the facilitation and monitoring of proper folding by chaperone interactions and the formation of assemblies into multimeric proteins in the endoplasmic reticulum. In the metabolism category, most DEGs were involved in “carbon metabolism”, followed by “glycolysis/gluconeogenesis” and “starch and sucrose metabolism”, suggesting that carbon and energy supplies were very important for the adaptation of *M. falcata* to low temperatures. In the cellular process category, most DEGs functioned in endocytosis. Endocytosis regulates the entry of membrane proteins, lipids, and extracellular molecules into the cell under adverse environmental conditions and plays a key role in alleviating ROS [45, 46].

### Genes encoding TFs in response to low-temperature stress

TFs play an important role in cell function and development and directly regulate gene expression via interactions with themselves and proteins to participate in plant stress regulatory processes, including low-temperature-related processes. Many TFs, including bHLH, bZIP, MYB, C2H2, ERF, NAC and WRKY types, that confer low-temperature tolerance to plants have been identified via transcriptomic approaches [47]. In this study, 1538 TF genes were differentially expressed between different temperature points and were classified into 45 TF gene families. The most abundant TF family was the WRKY family, followed by the ERF, MYB, bHLH and NAC families, and the dynamic changes in gene expression associated with these TFs may reveal their vital function in *M. falcata* low-temperature tolerance. Our results are consistent with those of previous reports on TFs involved in plant responses to low-temperature stress [31, 36] and suggest that members of the WRKY family play a critical role in *M. falcata* low-temperature tolerance.

### Identification of genes responsible for the response to low temperature

Plants have developed many mechanisms and pathways that enable them to minimize the negative effects of low temperature. Global analysis of stress-responsive genes facilitates the understanding of plant responses to low-temperature stress. In this paper, a total of 8683 DEGs were identified by a comparison of each temperature point to the control environment, and 134 genes, including 101 whose expression was upregulated and 33 whose expression was downregulated, were determined to be differentially coexpressed at all five temperature points (Fig. 4). However, only 7 genes were successfully annotated via GO enrichment analysis, and two genes (PB40804 and PB75011) were enriched in all three categories. The expression of both of these genes was upregulated under low-temperature stress. The product of PB40804 functions as a phenylalanine ammonia-lyase, which acts as a smart switch directly controlling the accumulation of calycosin and calycosin-7-O-beta-D-glucoside in *Astragalus membranaceus* plants during low-temperature treatment [48]. The PB75011 protein functions as a decarboxylase and is involved in cell wall/membrane/envelope biogenesis. Ornithine decarboxylase and arginine decarboxylase control the synthesis of polyamines in plants. The response of *Arabidopsis thaliana* to low-temperature stress emphasizes the involvement of transcriptional regulation in arginine decarboxylase gene expression [49]. On the basis of the COG annotation, we identified 18 enriched genes, including 15 whose expression was upregulated and 3 whose expression was downregulated. PB40804 was also annotated as “amino acid transport and metabolism” according to the COG analysis. The abundance of amino acid transporters is correlated with a multitude of fundamental roles in plant growth and development, and low-temperature stress could decrease the amino acid concentrations and alter their composition [50, 51]. “Circadian rhythm - plant” was the most enriched pathway according to the KEGG results, and we found that PB110405, the gene associated with the most GO terms in the biological process category, was enriched in this pathway. PB110405 was annotated with 9 GO terms in the biological process category, including “regulation of transcription, DNA-templated”, “temperature compensation of the circadian clock”, “response to hydrogen peroxide”, “starch metabolic process” and “response to cold”. The association with the “circadian rhythm-plant” pathway suggested that the internal temperature of *M. falcata* was substantially influenced by low temperature. The expression of PB108808, a putative ortholog of a MYB-related LHY TF gene in *Arabidopsis*, is a gene that is induced by low temperature and that indicates the presence of interplay between the circadian rhythm and the response to low temperature in *M. falcata.* The next pathway was “arginine and proline metabolism”; arginine and proline metabolism is one of the central pathways for the biosynthesis of the amino acids arginine and proline [27]. Proline accumulation is a well-known means of alleviating abiotic stress in plants [52]. Combined with the changes in proline contents under low-temperature stress, our results indicated that osmotic regulatory substances, protective protein molecules in *M. falcata*, play important roles in the response to low-temperature stress, and the composition of aromatic compounds may change under low-temperature stress.

### Identification of genetic modules corresponding to low-temperature stress

WGCNA is a systematic biological method that can be applied to the study of biological processes with multiple sources [53]. It has been proven that WGCNA can be an efficient data mining method, specifically for screening genes related to traits and for conducting modular classification to determine coexpression modules with high biological significance [54]. In this paper, 8683 DEGs were identified by a comparison of low-temperature-treated samples and control samples and were clustered into 12 gene modules after the modules with similar expression patterns were merged together (Fig. 5). It was found that the MEbrown module was significantly correlated with low-temperature stress in *M. falcata*. GO enrichment analysis of the MEbrown module revealed that regulatory pathways with biological significance could be obtained in this module. For example, the GO annotation terms “cold-response pathways”, “response to stress” and “intracellular signal transduction” were enriched. COG classification revealed that the MEbrown module was enriched in many DEGs associated with general function prediction only and the signal transduction mechanism, and KEGG pathway enrichment analysis revealed that there were many DEGs involved in starch and sucrose metabolism. We also found that electrolyte leakage was correlated with more genetic modules than the other physiological indicators were, which corroborated our findings from physiological assays in that electrolyte leakage can be used as a direct stress marker of cell membrane damage from low-temperature stress.

## Conclusions

Overall, by combining SMRT and NGS technologies, we explored the transcriptomic changes in the roots of low-temperature-treated *M. falcata* plants. A total of 115,153 nonredundant sequences were obtained, and 8849 AS events, 73,149 SSRs and 4189 lncRNAs were predicted. A total of 111,587 genes from SMRT sequencing were annotated, and 11,369 DEGs were identified that are involved in plant hormone signal transduction, protein processing in the endoplasmic reticulum, carbon metabolism, glycolysis/gluconeogenesis, starch and sucrose metabolism, and endocytosis pathways. We characterized 1538 TF genes into 45 TF gene families, and the most abundant TF family was the WRKY family, followed by the ERF, MYB, bHLH and NAC families. A total of 134 genes were differentially coexpressed at all five temperature points, including 101 genes whose expression was upregulated and 33 genes whose expression was downregulated. PB40804, PB75011, PB110405 and PB108808 were found to play crucial roles in the tolerance of *M. falcata* to low temperature. The WGCNA results showed that the MEbrown module was significantly correlated with low-temperature stress in *M. falcata*. Moreover, electrolyte leakage was correlated with most genetic modules and verified that electrolyte leakage can be used as a direct stress marker of cell membrane damage from low-temperature stress in physiological assays. These findings provide a complete characterization of gene transcription and facilitate the understanding of the mechanisms of tolerance to low temperature in *M. falcata.*

## Methods

### Plant cultivation and low-temperature treatment

Seeds of *M. falcata* L. cv. Hulunbuir were collected from the Hulunbuir Grassland with the permission of the Hulunbuir Grassland Station in the Inner Mongolia Province of China. The Hulunbuir Grassland Station in the Inner Mongolia Province of China undertook the formal identification of the samples and provided details of specimen deposited. It is a cultivated variety approved by the National Grass Variety Approval Committee of China (Accession number: 269). Collection of the seeds needs the permission of the Hulunbuir Grassland Station in the Inner Mongolia Province of China. The experimental research on *M. falcata* complies with Chinese and international guidelines. Seeds of *M. falcata* were disinfected with 5% sodium hypochlorite solution for 5 min and then washed in distilled water. The seeds were then germinated on wet filter paper in culture dishes in a growth chamber at 25 °C in the dark. Five-day-old seedlings were transplanted into plastic pots that were filled with a mixture of vermiculite:perlite:peat (1:1:1) in the greenhouse, whose average temperature was 25 °C and 20 °C and whose relative humidity was 55 and 70% during the day and night, respectively. All the seedlings were watered with 1/2-strength Hoagland [55] nutrient solution every two days. Ninety days after transplantation, uniform seedlings were transported to a growth chamber for the low-temperature treatment. The treatment temperatures were 4 °C, 0 °C, − 5 °C, − 10 °C and − 15 °C; the normal environment temperature was used as a control. For temperatures below 0 °C (freezing damage), *M. falcata* seedlings were acclimated for 2 days at 4 °C and then exposed to low-temperature stress. The cold stress induction schedule involved a decrease of 1 °C every 1 h from 4 °C, and the low-temperature stress treatment was applied at each studied temperature for 2 h [56, 57]. The roots were harvested, immediately frozen in liquid nitrogen and stored at − 80 °C for laboratory analysis.

### Physiological assays of low-temperature-treated *M. falcata* roots

Root cell membrane damage was assessed via electrolyte leakage [58]. The MDA content was measured according to the modified thiobarbituric acid (TBA) method [59], and the activity of SOD was measured by the nitro blue tetrazolium (NBT) method [60]. The activity of CAT was measured according to the methods of Maehly and Chance [61], and the activity of POD was measured according to the methods of Zaharieva et al. [62]. The O_2_^.-^ contents were determined as described by Elstner [63], the soluble protein content was determined according to the Bradford method [64], the content of reduced GSH was fluorometrically estimated [65], the proline content was determined by the ninhydrin method [66], and the soluble sugar content was determined according to the methods of Dreywood [67].

All assays described above were repeated four times, with four biological replicates. The data, which are shown as the means ± SDs, were subjected to ANOVA to determine significant differences. The least significant differences (LSDs) of the means were determined via Duncan’s test at the level of significance defined as α = 0.05.

### RNA isolation, library preparation and sequencing

Total RNA from each sample was isolated via TRIzol reagent (Invitrogen, USA) according to the manufacturer’s protocol, and genomic DNA was removed via digestion with DNase I (TaKaRa, Japan). The purity, concentration and nucleic acid absorption peak were then measured with a Nanodrop ND-1000 spectrophotometer (NanoDrop, USA). The RNA integrity was measured by an Agilent 2100 Bioanalyzer (Agilent, USA), and genomic DNA contamination was detected by electrophoresis.

The library was prepared after the samples passed the quality tests. For Illumina cDNA library preparation, 20 μg of total RNA from each pool was enriched with oligo (dT) magnetic beads and randomly interrupted by the addition of fragmentation buffer. First-strand cDNA was then synthesized with random hexamers, with mRNA used as a template. Second-strand cDNA was synthesized after the addition of buffer, dNTPs, RNase H and DNA polymerase I. The cDNA was subsequently purified with AMPure XP beads. The purified double-stranded cDNA was subjected to end repair, the addition of a poly-A tail and ligation with sequencing linkers, and the fragment size was selected via AMPure XP beads. Finally, the cDNA library was prepared by PCR-based enrichment.

With respect to PacBio Iso-Seq library preparation, the cDNA was synthesized using a SMARTer™ PCR cDNA Synthesis Kit (TaKaRa, Japan). cDNA libraries of different sizes were generated by BluePippin. The screened cDNA was then amplified by PCR, end repaired, connected to the SMRT dumbbell-type connector, and exonuclease digested. Finally, the library was prepared after a secondary screening by BluePippin. A total of eight SMRT cells were used for the three libraries at three size ranges: 1–2 kb, 2–3 kb, and 3–6 kb.

After the accurate quantification of libraries via Qubit 2.0 and qualification of the library sizes via an Agilent 2100 instrument, the libraries were sequenced via PacBio RS II (with 8 SMRT cells) and via the Illumina HiSeq 2500 platform at the Biomarker Institute (Biomarker, China). The 1–2 kb, 2–3 kb and 3–6 kb libraries were sequenced in conjunction with 3, 3 and 2 SMRT cells, respectively.

### Quality filtering and transcriptome assembly

Raw reads were processed into error-corrected ROIs by the ToFu pipeline, with full passes> = 0, and the accuracy of the sequence was greater than 0.75 (https://github.com/PacificBiosciences/cDNA_primer/wiki/Understanding-PacBio-transcriptome-data#readexplained). High-quality clean data were obtained by removing reads containing connectors and low-quality reads (including those with an N removal ratio greater than 10% and reads where the number of bases with a mass value of Q ≤ 10 accounted for more than 50% of the reads). FLNC transcripts were then determined by searching for poly-A tail signals and the 5′ and 3′ cDNA primers in ROIs. Iterative clustering for error correction (ICE) was used to obtain consensus isoforms, and the full-length consensus sequences from ICE were polished using SMRT Analysis (version 2.3.0). Full-length transcripts with a post correction accuracy greater than 99% were generated for further analysis. Redundant reads were removed from the Iso-Seq™ high-quality full-length transcripts by CD-HIT (identity > 0.99). The resulting transcript sequence was directly used for subsequent analyses of AS events, SSRs and lncRNAs. The second-generation data were used to quantify and differentially analyze the new CDS.

### Identification of AS events, SSRs, lncRNAs and CDSs

The Iso-Seq™ data was used to perform all-vs-all BLAST, and the BLAST alignments met all criteria were considered as the products of candidate AS events. The AS gap was larger than 100 bp and at least 100 bp away from the 3′/5′ end. SSRs within the transcriptome were identified by MISA (http://pgrc.ipk-gatersleben.de/misa/). Transcripts with lengths greater than 200 nt and with more than two exons were selected as lncRNA candidates and further screened via CPC/CNCI/CPAT/Pfam, which have the power to distinguish protein-coding genes from the noncoding genes. TransDecoder (https://github.com/TransDecoder/TransDecoder/releases) was used to identify CDS regions within the transcript sequences.

### Gene functional annotation

To acquire the most comprehensive annotation information, all full-length transcripts identified from the SMRT sequencing were aligned with the NCBI Nr, SwissProt, GO, COG, KOG, Pfam, and KEGG databases by BLAST software (version 2.2.26) [68]. GO enrichment analysis was implemented by the GOseq R package on the basis of Wallenius noncentral hypergeometric distribution [69]. KOBAS software was subsequently used to test the statistical enrichment of DEGs in the KEGG pathways [70].

### Quantification of gene expression levels

The gene expression level of each sample was identified by RSEM [71]. The clean data were mapped back onto the assembled transcriptome, and read count for each gene was obtained from the mapping results. Bowtie2 software was used to compare the clean data from Illumina sequencing to the SMRT sequencing data. Quantification of gene expression levels was estimated via FPKM values, considering the effects of the sequencing depth and gene length on the fragments.

### Identification of DEGs

Differential expression analysis was performed by the DESeq R package (version 1.10.1) to identify DEGs between the low-temperature-treated samples and the control samples collected at different temperature points [72]. DESeq performs statistical analyses for determining differential expression among digital gene expression data via a model based on a negative binomial distribution. For the detection of DEGs, a fold change≥2 and an FDR < 0.01 were used as screening criteria. The fold change represents the ratio of expression between two samples (groups). The FDR was obtained by correcting the *p* values of different significance. Because the differences in the transcriptome sequencing expression analysis are the transcribed expression values of a large number of independent statistical hypothesis tests, false positives are a concern; thus, in the process of analyzing DEGs, the recognized Benjamini-Hochberg correction method of hypothesis testing with the original significant *p* values for correction and, subsequently, the FDR were used as key indicators for screening DEGs.

### Identification of putative TFs

BLAST was used to search all the DEGs against plant a TF database (Plant TFDB version 4.0, http://planttfdb.cbi.pku.edu.cn) to identify putative TFs. The TF information was annotated on the basis of the comparison results.

### Coexpression network analysis with WGCNA

Coexpression networks were constructed via the WGCNA package in R from all the DEGs [53]. Modules were obtained by the automatic network construction function blockwise modules, with the default settings. The eigengene value was calculated for each module and used to test the association with each physiological index. The total connectivity and intramodular connectivity (function soft connectivity), kME (for modular membership), and kME *p* values were calculated for the DEGs.

### Validation of RNA-seq data by qRT-PCR

The RNA samples isolated above were used as templates and were reverse transcribed with a HiScript II Q Select RT SuperMix for qPCR (gDNA eraser) kit (Vazyme, China). Primers used in this study were designed via Primer 5 with RefSeq and are listed in Additional file 16: Table S11. The expression of the *beta-actin* gene was used as an internal control. qRT-PCR was performed with ChamQ™ Universal SYBR qPCR Master Mix (Vazyme, China) on a LightCycler 480 II58 device (Roche, Switzerland) according to the manufacturers’ protocol. Relative gene expression levels were evaluated according to the 2^-ΔΔCT^ method [73].

## Supplementary information

**Additional file 1: Table S1**. Overview of the quality of the sequence data obtained by NGS sequencing.

**Additional file 2: Table S2**. Statistics of AS. The QueryName and SubjectName are the IDs of the identified AS events. QhspStart1, QhspEnd1, QhspStart2, QhspEnd2, ShspStart, ShspEnd1, ShspStart2 and ShspEnd2 indicate the start and end positions of the 2 HSPs for these two AS events.

**Additional file 3: Table S3**. Statistics of SSRs.

**Additional file 4: Fig. S1**. Venn diagram of lncRNAs via CPC, CNCI, CPAT and information in the Pfam database.

**Additional file 5: Table S4**. Statistics of annotated transcripts.

**Additional file 6: Fig. S2**. Nr homologous species distribution.

**Additional file 7: Table S5**. Summary of reads from the RNA-seq data and their matches with full-length transcripts.

**Additional file 8: Table S6**. FPKM values and functional annotations of all transcripts.

**Additional file 9: Table S7**. Information on the 11,369 genes differentially expressed under low-temperature stress.

**Additional file 10: Table S8**. Summary of the 1538 TFs in response to low-temperature stress.

**Additional file 11: Fig. S3**. Expression pattern of TFs. The heat map shows the log_10_(FPKM+ 1) values of 1538 individual TFs.

**Additional file 12: Table S9**. Summary of coexpressed genes between low-temperature-treated samples and control samples.

**Additional file 13: Table S10**. Genetic modules and kME values of genes identified in the WGCNA results.

**Additional file 14: Fig. S4**. Functional analysis of genes in the MEbrown module. A, COG functional classification. B, GO classification. C, KEGG enrichment.

**Additional file 15: Fig. S5**. qRT-PCR assay results of genes selected for RNA-seq data confirmation. The blue line represents the normalized expression (log_10_(FPKM+ 1)) of RNA-seq data shown on the Y-axis on the left. The red line represents the relative qRT-PCR expression level data shown on the Y-axis on the right.

**Additional file 16: Table S11**. List of primers used in this paper.

## Data Availability

All relevant supplementary data are provided within this manuscript as Additional files. The PacBio SMRT reads and the Illumina NGS reads generated in this study were submitted to the NCBI Sequence Read Archive under accession numbers BioProject PRJNA 549099 and 520970, respectively. The address is as follows: http://www.ncbi.nlm.nih.gov/sra.

## Abbreviations

AS: Alternative splicing
CAT: catalase
CDS: coding DNA sequence
CNCI: Coding–non-coding index
COG: Clusters of Orthologous Groups
COLD1: Chilling-tolerance divergence 1
COR: Cold-responsive
CPAT: Coding potential assessment tool
CPC: Coding potential calculator
DEG: Differentially expressed gene
FDR: False discovery rate
FLNC: full-length nonchimeric read
FPKM: Fragments per kilobase of transcript sequence per million base pairs sequenced
GO: Gene Ontology
GSH: Reduced glutathione
ICE1: inducer of CBF expression 1
KEGG: Kyoto Encyclopedia of Genes and Genomes
KOG: Eukaryotic Ortholog Groups
LEA: Late embryogenesis abundant proteins
lncRNA: Long noncoding RNA
LSD: least significant difference
MDA: Malondialdehyde
NGS: Next-generation sequencing
Nr: NCBI nonredundant protein
O_2_^.-^: Superoxide anion radical
Pfam: Protein family
POD: Peroxidase
qRT-PCR: Quantificational real-time polymerase chain reaction
RNA-seq: RNA sequencing
ROI: Read of inserts
ROS: Reactive oxygen species
RSEM: RNA sequencing by expectation maximization
SMRT: Single-molecule real-time
SOD: Superoxide dismutase
SPS: Sucrose phosphate synthase
SSR: Simple sequence repeat
TF: Transcription factor
WGCNA: Weighted gene coexpression network analysis
