# Supplementary material for: Full-length transcriptome sequencing reveals the low-temperature-tolerance mechanism of Medicago falcata roots
Source: BMC Plant Biol. 2019 Dec 21;19:575. doi: 10.1186/s12870-019-2192-1 (PMC6925873; doi:10.1186/s12870-019-2192-1)
Supplement: Supplementary file 1 — Additional file 1: Table S1. Overview of the quality of the sequence data obtained by NGS sequencing. [file 12870_2019_2192_MOESM1_ESM.docx]

**Additional file 1: Table S1.** Overview of the quality of the sequence data obtained by NGS sequencing.

| Samples | Sample ID | Read Number | Base Number | GC (%) | Q30 (%) |
| --- | --- | --- | --- | --- | --- |
| CK-1 | T01 | 23091037 | 6927827338 | 42.68 | 87.05 |
| CK-2 | T02 | 20737440 | 6228623630 | 43.07 | 85.78 |
| CK-3 | T03 | 24821593 | 7452960042 | 43.80 | 87.62 |
| 4 ℃-1 | T04 | 23682406 | 7111318530 | 42.32 | 87.15 |
| 4 ℃-2 | T05 | 22622376 | 6788345046 | 42.81 | 85.76 |
| 4 ℃-3 | T06 | 22076902 | 6634068078 | 42.36 | 85.64 |
| 0 ℃-1 | T07 | 23376615 | 7013909552 | 42.28 | 87.20 |
| 0 ℃-2 | T08 | 23981321 | 7198572716 | 43.34 | 87.77 |
| 0 ℃-3 | T09 | 21841610 | 6557357894 | 42.15 | 85.71 |
| -5 ℃-1 | T10 | 23825136 | 7156504880 | 42.16 | 87.59 |
| -5 ℃-2 | T11 | 25084542 | 7514124956 | 42.54 | 87.77 |
| -5 ℃-3 | T12 | 22361195 | 6703474438 | 42.78 | 85.57 |
| -10 ℃-1 | T13 | 27468972 | 8221765030 | 43.31 | 92.71 |
| -10 ℃-2 | T14 | 26079064 | 7804333894 | 42.16 | 92.38 |
| -10 ℃-3 | T15 | 21727414 | 6491392924 | 42.28 | 93.33 |
| -15 ℃-1 | T16 | 21133043 | 6321295340 | 41.92 | 85.73 |
| -15 ℃-2 | T17 | 21085644 | 6340589912 | 41.87 | 85.67 |
| -15 ℃-3 | T18 | 23499333 | 7008671796 | 43.08 | 85.51 |
| Total/average |  | 418495643 | 125475135996 | 42.61 | 87.55 |
