# Supplementary material for: Full-length transcriptome sequencing reveals the low-temperature-tolerance mechanism of Medicago falcata roots
Source: BMC Plant Biol. 2019 Dec 21;19:575. doi: 10.1186/s12870-019-2192-1 (PMC6925873; doi:10.1186/s12870-019-2192-1)
Supplement: Supplementary file 5 — Additional file 5: Table S4. Statistics of annotated transcripts. [file 12870_2019_2192_MOESM5_ESM.docx]

**Additional file 5: Table S4.** Statistics of annotated transcripts.

| Anno_Database | Annotated_Number | 300 bp <=length<1000 bp | length>=1000 bp |
| --- | --- | --- | --- |
| COG_Annotation | 44099 | 2310 | 41789 |
| GO_Annotation | 3385 | 212 | 3173 |
| KEGG_Annotation | 44974 | 2844 | 42130 |
| KOG_Annotation | 66471 | 3662 | 62809 |
| Pfam_Annotation | 89117 | 5147 | 83970 |
| Swissprot_Annotation | 82433 | 4732 | 77701 |
| eggNOG_Annotation | 104568 | 6352 | 98216 |
| nr_Annotation | 111384 | 7113 | 104271 |
| All_Annotated | 111587 | 7155 | 104432 |
