# Supplementary material for: Full-length transcriptome sequencing reveals the low-temperature-tolerance mechanism of Medicago falcata roots
Source: BMC Plant Biol. 2019 Dec 21;19:575. doi: 10.1186/s12870-019-2192-1 (PMC6925873; doi:10.1186/s12870-019-2192-1)
Supplement: Supplementary file 7 — Additional file 7: Table S5. Summary of reads from the RNA-seq data and their matches with full-length transcripts. [file 12870_2019_2192_MOESM7_ESM.docx]

**Additional file 7: Table S5.** Summary of reads from the RNA-seq data and their matches with full-length transcripts.

| Sample | Total Reads | Mapped Reads (%) | Uniq mapped Reads (%) | Multi mapped Reads (%) |
| --- | --- | --- | --- | --- |
| CK-1 | 23,091,037 (100%) | 17,490,306 (75.75%) | 2,022,351 (11.56%) | 15,467,955 (88.44%) |
| CK-2 | 20,737,440 (100%) | 15,806,235 (76.22%) | 1,693,674 (10.72%) | 14,112,561 (89.28%) |
| CK-3 | 24,821,593 (100%) | 18,665,971 (75.20%) | 1,977,381 (10.59%) | 16,688,590 (89.41%) |
| 4-1 | 23,682,406 (100%) | 18,481,478 (78.04%) | 1,804,742 (9.77%) | 16,676,736 (90.23%) |
| 4-2 | 22,622,376 (100%) | 17,930,456 (79.26%) | 1,676,166 (9.35%) | 16,254,290 (90.65%) |
| 4-3 | 22,076,902 (100%) | 17,319,944 (78.45%) | 1,739,251 (10.04%) | 15,580,693 (89.96%) |
| 0-1 | 23,376,615 (100%) | 17,848,326 (76.35%) | 1,852,673 (10.38%) | 15,995,653 (89.62%) |
| 0-2 | 23,981,321 (100%) | 18,592,191 (77.53%) | 1,736,659 (9.34%) | 16,855,532 (90.66%) |
| 0-3 | 21,841,610 (100%) | 16,811,591 (76.97%) | 1,700,460 (10.11%) | 15,111,131 (89.89%) |
| -5-1 | 23,825,136 (100%) | 18,373,303 (77.12%) | 1,911,314 (10.40%) | 16,461,989 (89.60%) |
| -5-2 | 25,084,542 (100%) | 19,339,663 (77.10%) | 1,944,301 (10.05%) | 17,395,362 (89.95%) |
| -5-3 | 22,361,195 (100%) | 17,390,612 (77.77%) | 1,766,868 (10.16%) | 15,623,744 (89.84%) |
| -10-1 | 27,468,972 (100%) | 20,608,468 (75.02%) | 1,901,060 (9.22%) | 18,707,408 (90.78%) |
| -10-2 | 26,079,064 (100%) | 19,248,034 (73.81%) | 1,982,092 (10.30%) | 17,265,942 (89.70%) |
| -10-3 | 21,727,414 (100%) | 16,881,193 (77.70%) | 1,714,623 (10.16%) | 15,166,570 (89.84%) |
| -15-1 | 21,133,043 (100%) | 16,446,758 (77.82%) | 1,693,986 (10.30%) | 14,752,772 (89.70%) |
| -15-2 | 21,085,644 (100%) | 16,177,861 (76.72%) | 1,751,615 (10.83%) | 14,426,246 (89.17%) |
| -15-3 | 23,499,333 (100%) | 18,210,829 (77.50%) | 1,743,391 (9.57%) | 16,467,438 (90.43%) |
