# Supplementary figures and images for: Full-length transcriptome sequencing reveals the low-temperature-tolerance mechanism of Medicago falcata roots
Source: BMC Plant Biol. 2019 Dec 21;19:575. doi: 10.1186/s12870-019-2192-1 (PMC6925873; doi:10.1186/s12870-019-2192-1)

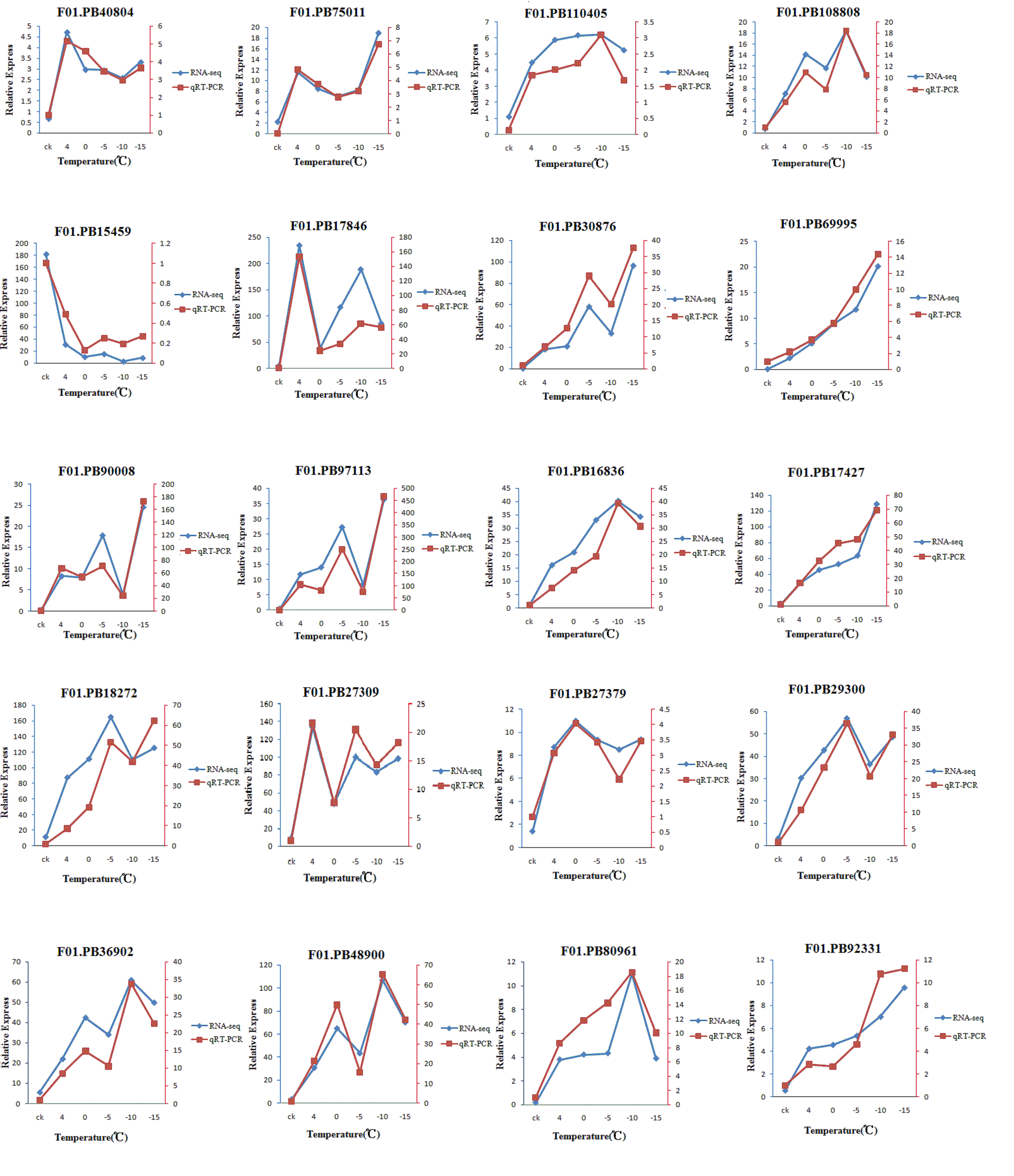

Supplement: Supplementary file 15 — Additional file 15: Fig. S5. qRT-PCR assay results of genes selected for RNA-seq data confirmation. The blue line represents the normalized expression (log10(FPKM+ 1)) of RNA-seq data shown on the Y-axis on the left. The red line represents the relative qRT-PCR expression level data shown on the Y-axis on the right. [file 12870_2019_2192_MOESM15_ESM.tif]
