# Supplementary material for: Full-length transcriptome sequencing reveals the low-temperature-tolerance mechanism of Medicago falcata roots
Source: BMC Plant Biol. 2019 Dec 21;19:575. doi: 10.1186/s12870-019-2192-1 (PMC6925873; doi:10.1186/s12870-019-2192-1)
Supplement: Supplementary file 16 — Additional file 16: Table S11. List of primers used in this paper. [file 12870_2019_2192_MOESM16_ESM.docx]

**Additional file 16: Table S11.** List of primers used in this paper.

| Gene IDs | Forward primer（5’-3’） | Reverse primer（5’-3’） |
| --- | --- | --- |
| F01.PB40804 | GGAAACCCAACAATCCCAAAC | CTCCGGTTAGTAAACCAGTTCC |
| F01.PB75011 | TCGTCCAGCTGCAATCTATG | TCTGAACAGAAGCAGACCTAAC |
| F01.PB110405 | TGAGGACTTGCACACAATATCA | GCACAAACAGCAGCCATAAC |
| F01.PB108808 | ACCTGATGCGGACCATTTAG | TACCTCTTCAGACACCTCCTT |
| F01.PB15459 | GTCGTGCTGGTCTCATCAA | GACCCACTGACCCGTAAATAG |
| F01.PB17846 | CGGCTTGTACCGGTGTTTAT | CAATCGCAGGCAGCAAATAC |
| F01.PB30876 | CTCAGCAAGTGCCTGACTTTA | CCTCTGTGAACCATTCCATCTC |
| F01.PB69995 | GCATTTCAGGATCAGTGTCTTC | CTGCAACAGCTCCAATCTTC |
| F01.PB90008 | CCAGGACAAAGCTCCTAACAA | GTTCCTCCGGAAGTCCAATATC |
| F01.PB97113 | CCAGGACAAAGCTCCTAACAA | GTTCCTCCGGAAGTCCAATATC |
| F01.PB16836 | GTCGTGTACCGTACCTTTGTT | CAACTTCCTCCTCGTTCTCATC |
| F01.PB17427 | GTCGTGTACCGTACCTTTGT | CCTTAACTTCCTCCTCGTTCTC |
| F01.PB18272 | GTCGTGTACCGTACCTTTGT | CCTTAACTTCCTCCTCGTTCTC |
| F01.PB27309 | GTTGCAACGGGAATGTTGTG | CCTTCTTCCTCTGATCACCTTG |
| F01.PB27379 | GGAGCTTACATCGTGAGACAAG | GGACACCGATAGCATAGGAAAC |
| F01.PB29300 | GTCGTGTACCGTACCTTTGT | CCTTAACTTCCTCCTCGTTCTC |
| F01.PB36902 | GGACCCAAATACGAGTAGAGATG | GCCACTGTCTTCCCATAACA |
| F01.PB48900 | ACCTGATGCGGACCATTTAG | GCAACACCTCTCTGGAGAATAG |
| F01.PB80961 | ACCTGATGCGGACCATTTAG | GCAACACCTCTCTGGAGAATAG |
| F01.PB92331 | CCAGCAGTGAATGGAACAAAG | TAGACCGTTCCAATCGATGAAG |
| β-*actin* | TTTGAGACTTTCAATGTGCCCGCC | TAGCATGTGGGAGTGCATAACCCT |
